# Privacy-Preserving Self-Helped Medical Diagnosis Scheme Based on Secure Two-Party Computation in Wireless Sensor Networks

**DOI:** 10.1155/2014/214841

**Published:** 2014-07-14

**Authors:** Yi Sun, Qiaoyan Wen, Yudong Zhang, Wenmin Li

**Affiliations:** ^1^State Key Laboratory of Networking and Switching Technology, Beijing University of Posts and Telecommunications, Beijing 100876, China; ^2^Brain Image Processing, Columbia University, New York, NY 10032, USA

## Abstract

With the continuing growth of wireless sensor networks in pervasive medical care, people pay more and more attention to privacy in medical monitoring, diagnosis, treatment, and patient care. On one hand, we expect the public health institutions to provide us with better service. On the other hand, we would not like to leak our personal health information to them. In order to balance this contradiction, in this paper we design a privacy-preserving self-helped medical diagnosis scheme based on secure two-party computation in wireless sensor networks so that patients can privately diagnose themselves by inputting a health card into a self-helped medical diagnosis ATM to obtain a diagnostic report just like drawing money from a bank ATM without revealing patients' health information and doctors' diagnostic skill. It makes secure self-helped disease diagnosis feasible and greatly benefits patients as well as relieving the heavy pressure of public health institutions.

## 1. Introduction

With the rapid development of science, more and more advanced technologies such as the internet of things and cloud computing are utilized in the area of modern medicine and this trend further pushes healthcare into the digital era [1–3]. Currently, numerous healthcare devices such as heart rate monitor, blood pressure monitor, and electrocardiogram are already popular in people's normal life. It makes it convenient for people to be aware of their health situation by viewing the reports of these devices. Especially, by the growing use of sensor technology in telecare, the new field known as wireless body area networks (WBAN) [1, 4] has designed various sensor devices that can be used to supervise critical body parameters and activities anytime and anywhere. People can easily and conveniently get the health data by these advanced sensor devices [5] such as temperature measurement, respiration monitor, heart rate monitor, pulse oximeter SpO2, blood pressure monitor, pH monitor, glucose sensor, cardiac arrhythmia monitor/recorder, brain liquid pressure sensor, and endoscope capsule. What is more, these devices are becoming more functional and portable. More and more mobile medical monitors have already been used to serve us [2].

Therefore, people no longer worry about how to obtain the health data but are concerned about how to securely deal with these sensitive data to have disease diagnosis with a medical institution. Traditionally, the issue of privacy of medical data has been dealt with primarily as a policy problem [6, 7]. Many related laws have been issued to protect the privacy of patients. However, it is still far away from satisfactory and people still fear the leakage of their private data. Hence, the most efficient solution to this problem is to protect patients' privacy in technology rather than in policy alone. In this aspect, most of previous literatures have introduced homomorphic encryption (HE) [8–10] to protect patients' privacy in some privacy-preserving medical applications [11]. However, HE will inevitably introduce tremendous cost and is not applicable to practical large-scale applications. Therefore, in this paper, we focus on building a secure and practical privacy-preserving medical diagnosis system that can serve us in our daily life. Starting from the aspiration of the patient, the most secure and plausible diagnostic method is to apply the processed data rather than the original data to interact with the hospital which owns a disease database to diagnose the health status privately. Moreover, it requires that after diagnosis, the hospital gets nothing about the patient's health data and the patient has no idea of the hospital's disease database.

Inspired by daily used bank automated teller machine (ATM), we introduce the privacy-preserving self-helped medical diagnosis ATM (MD-ATM) so that after obtaining a healthcare card that stores some information about the health data which is collected by various sensor medical devices, patients can privately diagnose himself by inserting the health card into the MD-ATM to obtain diagnostic report just like drawing money from a bank ATM without revealing patient's health information and the disease database or doctors' diagnostic skill. When needing local computing, storing, or inputting some information, the patient uses his own portable device, called portable medical diagnostic device (PMDD).

In this paper, we will show how to realize this modern diagnosis system without HE. The main idea and technology we used in this scheme are secure two-party computation (STC) and oblivious transfer (OT). Firstly, we assume that patients themselves collect related data by various wireless sensor medical devices and further process and store them in their own health cards using PMDD. When diagnosing, the patient firstly transforms the original data locally and then inserts the card into the MD-ATM of the hospital to check up his health. Operating following the instructions of the MD-ATM, the patient will finally obtain a diagnostic report through OT and the patient then completes the self-helped diagnosis. In brief, our main contributions can be summarized as follows.

*Our Contributions.*

- We build a new “patient-centered” medical diagnosis model in wireless sensor networks where patients themselves collect health data by various sensor medical devices while the hospital provides a disease database to help patients to complete disease diagnosis by themselves. Compared with traditional “doctor-centered” medical diagnosis model where patients have to depend on the doctor, our system is more appropriate especially when people pay more and more attention to privacy in wireless sensor networks.
- We firstly propose the privacy-preserving self-helped MD-ATM to construct a secure medical diagnosis scheme following the idea of STC. It makes secure self-helped medical diagnosis feasible and convenient just like drawing money from a bank ATM. It will greatly benefit patients as well as relieving the heavy pressure of public health institutions.
- We construct the self-helped medical diagnosis system based on OT without expensive HE. It provides us with another perspective to consider the problem of secure medical diagnosis for patients.

The rest of this paper is organized as follows. In Section 2, we briefly give an overview of secure two-party computation and oblivious transfer, and then we present our medical diagnosis system model in Section 3. In Section 4, we propose our privacy-preserving self-helped medical diagnosis scheme in detail and give a strict proof based on real-ideal simulation paradigm in Section 5. Finally, we summarize our work of this paper in the last section.

## 2. Preliminaries

### 2.1. Secure Two-Party Computation

Secure multiparty computation (SMC) is dedicated to deal with the problem of secure computation among distrustful participants. It was first introduced by Yao in 1982 [12] and then was extended by Goldreich et al. [13] and many other researchers [14–19]. Generally speaking, SMC is a method to implement cooperative computation with participants' private data, ensuring the correctness of the computation as well as not disclosing additional information except the necessary results. It has become a research focus in the international cryptographic community due to its wide applications in various areas and a mass of research results have been published one after another. Secure two-party computation (STC) [20] is a special case in SMC where there are only two participants. The well-known millionaires' problem [12] put forward by Yao is the representative problem of STC. In our discussing, we will consider the two-party case.

Generally speaking, STC is dedicated to computing a certain function between two mutually distrusted participants on their private inputs without revealing their private information. Informally, assuming that there are 2 participants, *P*_1_, *P*_2_, each of them has a private number, *x*_1_, *x*_2_, respectively. They want to cooperate to compute the function *y* = *f*(*x*_1_, *x*_2_). A STC protocol is dubbed secure if no participant can learn more from the description of the public function and the result of the global calculation than what he can learn from his own information.

Formally, we usually analyze the security of a STC protocol using the real-ideal paradigm in the semihonest model where both of the two parties act semihonestly, following the protocol but making effort to gain more information about other parties' inputs, intermediate results, or overall outputs by the transcripts of the protocol [15]. We can overview the real-ideal paradigm as follows.

Firstly, in the ideal world, we assume that the computation of the functionality *F* on users private inputs is conducted by an additional trusted party, who receives *x*_*i*_ from user *P*_*i*_, *i* = 1,2, and returns the result *f*(*x*_1_, *x*_2_) to *P*_*i*_, *i* = 1,2. However, there is no trusted party in the real world and so the two parties have to run a protocol Π to get the desired result. During executing protocol Π, both parties act semihonestly. Herein, the view of the *i*th party during an execution of Π on *x*_1_, *x*_2_ is denoted as VIEW_*i*_^Π^(*x*_1_, *x*_2_), which contains *P*_*i*_'s input, random tape, and the messages received from the other party. For a deterministic private function *f*, we say that Π privately computes *f* if there exist probabilistic polynomial-time algorithms *S*_1_, *S*_2_, such that the simulated distribution {*S*_*i*_(*x*_*i*_; *f*(*x*_1_, *x*_2_))} is indistinguishable to {VIEW_*i*_^Π^(*x*_1_, *x*_2_)}, *i* = 1,2. That is,

(1) $$\begin{array}{c} \left\{S_{1}\left(x_{1};f\left(x_{1},x_{2}\right)\right)\right\}≅\left\{{\text{VIEW}}_{1}^{\Pi }\left(x_{1},x_{2}\right)\right\},\\ \left\{S_{2}\left(x_{2};f\left(x_{1},x_{2}\right)\right)\right\}≅\left\{{\text{VIEW}}_{1}^{\Pi }\left(x_{1},x_{2}\right)\right\}. \end{array}$$

### 2.2. Oblivious Transfer

In cryptography, OT is a type of protocol in which a sender transfers one of potentially many pieces of information to a receiver but remains oblivious as to which piece has been transferred. It was firstly introduced by Rabin [21] in 1981. Therein, the sender sends a message to the receiver with probability 1/2, while the sender remains oblivious as to whether or not the receiver received the message. Rabin's oblivious transfer scheme is based on the RSA cryptosystem. In 1985, Even et al. [22] proposed a more useful OT called 1-out-of-2 OT (OT_1_^2^) to build protocols for secure multiparty computation.

Afterwards, it has been generalized to 1-out-of-*m* OT (OT_1_^*m*^) [23] where the receiver gets exactly one message without the sender getting to know which message was queried and the receiver getting to know anything about the other messages that were not retrieved. OT_1_^*m*^ has become a fundamental tool in cryptography and is usually used as a black-box when constructing protocols.

Formally, we can describe an OT_1_^*m*^ protocol as follows. There are 2 participants called the sender *S* and the receiver *R*. Specifically, *S* has *m* messages, and *R* has an index *z*. *R* wishes to receive the *z*th message of the sender's *m* messages without leaking *z* to *S*, while knowing nothing about the rest *m* − 1 messages. A simplified OT_1_^*m*^ protocol can be presented as in Algorithm 1.

## 3. System Model

In this section, we present the system model including the goals we aim to achieve in detail.

In this paper, we consider the privacy-preserving medical diagnosis system with two participants: the patient and the hospital. We assume that each patient can collect his own health data such as heart beat and blood pressure, in the form of a vector, called query vector, easily by various advanced medical devices. Herein, we call the heart beat, blood pressure, and so forth, as parameter items and the health data corresponding to heart beat, blood pressure, and so forth, as parameter values. For example, *q*_*P*_ = (*q*_*P*1_,…, *q*_*Pn*_) is the query vector of the patient *P*, where all {*q*_*Pw*_}_*w*=1,…,*n*_ are the necessary parameters the hospital needs for diagnosis, and *q*_*Pw*_ is the parameter value of the parameter item heart beat. Each patient has a health card to store related data and a portable device PMDD to read the data stored in the card and to do some related computations after inserting the card. The hospital has a disease database DB = {*d*_*i*_}_*i*=1,…,*m*_, which in fact is the standard to determine which disease the patient has got. Each record of the disease database is presented as a triple *d*_*i*_ = (*i*, *t*_*i*_, *r*_*i*_), *i* = 1,…, *m*, where *m* is the capacity of the disease database; *i* is the index of a disease; *t*_*i*_, called the trait vector of the disease *d*_*i*_, is a vector that covers all necessary parameters the hospital needs for diagnosis; and *r*_*i*_ is the disease diagnostic report including the disease name, doctors' advices, and prescriptions corresponding to the *i*th disease *d*_*i*_. Concerning these parameters, we have some illustrations as follows.

- *q*_*P*_: it includes all necessary parameter items the hospital needs for diagnosis such as heart beat and blood pressure. The query vectors of the same patient *P* are different if *P* goes to different hospitals since their medical levels are different. The query vectors of the same patient *P* may be also different if *P* goes to the same hospital at different time since the medical level of the hospital has been always keeping improving. The dimension *n* and every parameter item {*q*_*Pw*_}_*w*=1,…,*n*_ of the query vector are determined by the trait vector *t*_*i*_ of the hospital. After registering to the hospital, patient can know what parameter items are needed in this diagnosis by reading the health card.
- DB = {*d*_*i*_}_*i*=1,…,*m*_: it concludes all diseases a hospital can diagnose. Different hospitals have different disease databases and the same hospital has different disease databases at different time since its medical level has been keeping improving. The dimension *m* is determined by the hospital's medical level.
- *t*_*i*_: it includes all necessary parameter items the hospital needs for diagnosis such as heart beat and blood pressure. Different hospitals have different trait vectors and the trait vectors of the same hospital at different time may be different since the medical level of the hospital has been always keeping improving. The dimension *n* and every parameter item {*t*_*ij*_}_*j*=1,…,*n*_ of the trait vector are determined by the hospital's medical level. In order to improve the precision of our diagnosis system, the hospital can consider as many factors as possible such as adding more personal feelings, symptoms, and previous medical features from the patient as parameter items. Although we only can diagnose some simple diseases currently, it is believed that it will be feasible for more complicated diseases in the future by extending the dimension *n* of the parameter items.
- *r*_*i*_: it includes the disease name, doctors' advices, and prescriptions corresponding to the *i*th disease *d*_*i*_. Each report may conclude many doctors' advices and prescriptions. Herein, we assume that every report obtained from the MD-ATM following the self-helped medical diagnosis is authorized by the hospital and all advices and prescriptions of a report are signed by corresponding doctors. After receiving the diagnostic report, patient can choose one doctor's advice and prescription to treat himself.

In this paper, the system makes medical diagnosis according to the Euclidean distances of two vectors. Specifically, taking the query vector of the patient and a trait vector of the database as an example, given a patient's query vector *q*_*P*_ = (*q*_*P*1_,…, *q*_*Pn*_) and a disease trait vector *t*_*i*_ = (*t*_*i*1_,…, *t*_*in*_), *i* ∈ {1,…, *m*}, their Euclidean distance [3] denoted by dist⁡_*q*_*P*_↔*t*_*i*__ is

(2) $$\begin{array}{c} {\mathrm{dist}}_{q_{P}↔t_{i}}=\sqrt{\overset{n}{\underset{w=1}{\sum }}{\left(q_{Pw}-t_{iw}\right)}^{2}}. \end{array}$$

Herein, we compare the squares of the Euclidean distances,

(3) $$\begin{array}{c} \text{dis}{\text{t}}_{q_{P}↔t_{i}}^{2}-\text{dis}{\text{t}}_{q_{P}↔t_{j}}^{2}=\overset{n}{\underset{w=1}{\sum }}{\left(q_{Pw}-t_{iw}\right)}^{2}-\overset{n}{\underset{w=1}{\sum }}{\left(q_{Pw}-t_{jw}\right)}^{2}. \end{array}$$

It is obvious that we can figure out which one has smaller distance with patient's query vector just by checking the sign of (3) without exact result of dist⁡_*q*_*P*_↔*t*_*i*__ or dist⁡_*q*_*P*_↔*t*_*j*__. Assuming that the report *r*_*z*_ corresponding to the trait vector *t*_*z*_, *z* ∈ {1,…, *m*}, is the diagnosed disease report, we have the following result, for all *j* = 1,…, *m*, *j* ≠ *z*:

(4) $$\begin{array}{c} \text{dis}{\text{t}}_{q_{P}↔t_{z}}^{2}-\text{dis}{\text{t}}_{q_{P}↔t_{j}}^{2}<0. \end{array}$$

In our scheme, we will compare the squares of the Euclidean distances of the query vector and the trait vectors to find the diagnostic report that satisfies (4).

**Figure 1:**
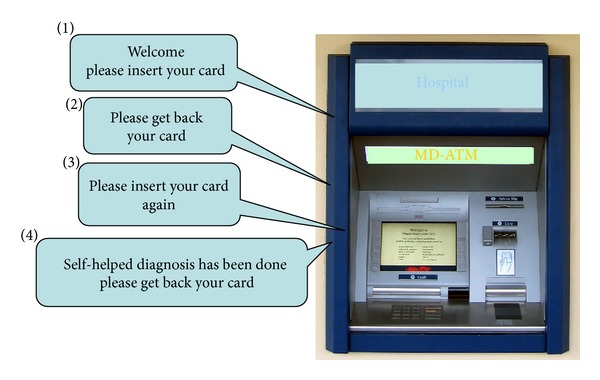
Self-helped medical diagnosis model of our scheme.

In real application, the hospital provides a MD-ATM, which is connected with the disease database and can read the data of the card, to direct patients to complete self-helped disease diagnosis. Specifically, we assume that each patient registers to the hospital for the first time and gets a health card. The hospital provides a self-helped MD-ATM in public just like a bank ATM. Whenever *P* wants to have a diagnosis, inserting his health card into the MD-ATM and following the instructions, *P* can complete the self-helped diagnosis by himself. The basic model can be illustrated in Figure 1.

Apart from the above, to enable a privacy-preserving medical diagnosis system, our scheme should simultaneously fulfill the following two security goals.

- Confidentiality of disease database should be protected during the self-helped diagnosis process.
- Confidentiality of patient's private health data should be protected during the self-helped diagnosis process.

## 4. Our Scheme

**Figure 2:**
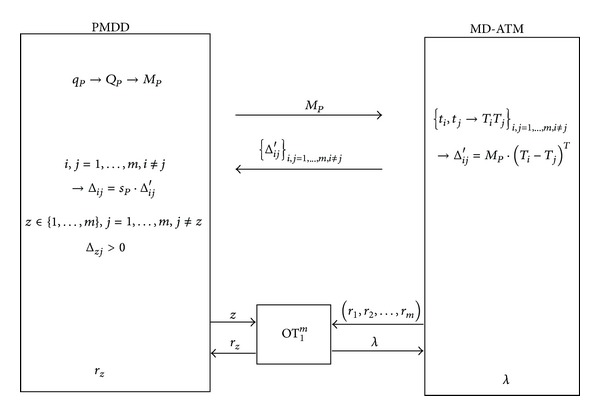
PP-SH-MDS.

In this section, we propose our privacy-preserving self-helped medical diagnosis scheme (PP-SH-MDS) in detail to show how a patient can diagnose by himself using his PMDD and the self-helped MD-ATM. The core of our construction can be summarized in Figure 2.

Specifically, the patient *P* executes as follows to make a self-helped diagnosis using his PMDD and the MD-ATM.

In the setup phase, *P* registers to a hospital as traditional medical diagnosis and gets a health card.

In the diagnosis phase, there are three subphases.

(*1) Local Preprocessing.* Whenever *P* wants to have a diagnosis, he firstly conducts the following two transformations on PMDD locally.

(i) Vector-to-Vector.
  (a) *P* firstly extends his original health data from an *n*-vector *q*_*P*_ = (*q*_*P*1_,…, *q*_*Pn*_) to an (*n* + 2)-vector *Q*_*P*_ = (*q*_*P*1_,…, *q*_*Pn*_, *q*_*P*,*n*+1_, *q*_*P*,*n*+2_), where *q*_*P*,*n*+1_ = −(1/2)∑_*w*=1_^*n*^ *q*_*Pw*_^2^ and *q*_*P*,*n*+2_ = 1.
(ii) Vector-to-Matrix.
  (a) *P* randomly chooses a password *s*_*P*_ = (*s*_*P*1_,…, *s*_*Pk*_) and then generates a *k* × (*n* + 2) matrix
    (5) $$\begin{array}{c} B_{P}=\left[\begin{array}{ccc} b_{11} & \cdots & b_{1,n+2}\\ \vdots & & \vdots \\ b_{k1} & \cdots & b_{k,n+2} \end{array}\right], \end{array}$$
   where ∑_*u*=1_^*k*^ *s*_*Pu*_ · *b*_*uw*_ = 1, *w* = 1,…, *n* + 2.
  (b) By blinding *Q*_*P*_ using the matrix *B*_*P*_, *P* further extends *Q*_*P*_ to a matrix
    (6) $$\begin{array}{c} M_{P}=\left[\begin{array}{ccc} b_{11}\cdot q_{P1} & \cdots & b_{1,n+2}\cdot q_{P,n+2}\\ \vdots & & \vdots \\ b_{k1}\cdot q_{P1} & \cdots & b_{k,n+2}\cdot q_{P,n+2} \end{array}\right]. \end{array}$$

After completing the above steps, *P* stores the matrix *M*_*P*_ in the health card.

*(2) Diagnosis.*

- After local preprocessing, *P* inserts his health card into the MD-ATM and then the MD-ATM reads the card to get the matrix *M*_*P*_ and randomly chooses two trait vectors *t*_*i*_ = (*t*_*i*1_,…, *t*_*in*_) and *t*_*j*_ = (*t*_*j*1_,…, *t*_*jn*_) and, respectively, extends them to (*n* + 2)-vectors *T*_*i*_ = (*t*_*i*1_,…, *t*_*in*_, *t*_*i*,*n*+1_, *t*_*i*,*n*+2_) and *T*_*j*_ = (*t*_*j*1_,…, *t*_*jn*_, *t*_*j*,*n*+1_, *t*_*j*,*n*+2_), where *t*_*i*,*n*+1_ = 1, *t*_*i*,*n*+2_ = −(1/2)∑_*w*=1_^*n*^ *t*_*iw*_^2^ and *t*_*j*,*n*+1_ = 1, *t*_*j*,*n*+2_ = −(1/2)∑_*w*=1_^*n*^ *t*_*jw*_^2^. Then the MD-ATM computes Δ_*ij*_′ = *M*_*P*_ · (*T*_*i*_−*T*_*j*_)^*T*^, *i*, *j* = 1,…, *m*, *i* ≠ *j*, and writes (Δ_*ij*_′, *i*, *j*) in the card and indicates the patient to get back his card.
- Following the instructions of the MD-ATM, the patient gets the card back and inserts it into PMDD. After inputting his password, PMDD begins to compute Δ_*ij*_ = *s*_*P*_ · Δ_*ij*_′, *i*, *j* = 1,…, *m*, *i* ≠ *j*, and finds the index *z* so that for all *j* = 1,…, *m*, *j* ≠ *z*,   Δ_*ij*_ > 0. *z* is the input of the following OT_1_^*m*^ protocol.

(*3) 1-out-of-m OT Protocol*.

- *P* inserts his card into the MD-ATM and invokes an OT_1_^*m*^ protocol, where *P*'s input is the index *z* and the MD-ATM's input is the diagnostic report set (*r*_1_, *r*_2_,…, *r*_*m*_) of the database.

After executing the OT_1_^*m*^ protocol, *P* gets the diagnostic report *r*_*z*_ corresponding to the disease *d*_*z*_ according to the index *z*, while the MD-ATM gets *Null*, denoted by *λ*.

## 5. Analysis

In this section, we analyze our scheme in detail. We firstly have a look at the correctness and then give a strict security proof following the real-ideal simulation paradigm of STC in the scenarios of semihonest adversaries.

### 5.1. Correctness

In this aspect, we follow the steps of our scheme and make sure that the patient indeed finds out the most possible disease from the disease database of the hospital using his health data by comparing Euclidean distances.

Following the scheme, we can see that the patient transforms the health data in two steps. Firstly, he extends his original health data from an *n*-vector *q*_*P*_ = (*q*_*P*1_,…, *q*_*Pn*_) to an (*n* + 2)-vector *Q*_*P*_ = (*q*_*P*1_,…, *q*_*Pn*_, *q*_*P*,*n*+1_, *q*_*P*,*n*+2_), where *q*_*P*,*n*+1_ = −(1/2)∑_*w*=1_^*n*^ *q*_*Pw*_^2^ and *q*_*P*,*n*+2_ = 1 and then blinds and extends *Q*_*P*_ using the matrix *B*_*P*_ to a matrix

(7) $$\begin{array}{c} M_{P}=\left[\begin{array}{ccc} b_{11}\cdot q_{P1} & \cdots & b_{1,n+2}\cdot q_{P,n+2}\\ \vdots & & \vdots \\ b_{k1}\cdot q_{P1} & \cdots & b_{k,n+2}\cdot q_{P,n+2} \end{array}\right]. \end{array}$$

On the other hand, the MD-ATM randomly selects two trait vectors *t*_*i*_ = (*t*_*i*1_,…, *t*_*in*_) and *t*_*j*_ = (*t*_*j*1_,…, *t*_*jn*_) and, respectively, extends them to (*n* + 2)-vectors *T*_*i*_ = (*t*_*i*1_,…, *t*_*in*_, *t*_*i*,*n*+1_, *t*_*i*,*n*+2_) and *T*_*j*_ = (*t*_*j*1_,…, *t*_*jn*_, *t*_*j*,*n*+1_, *t*_*j*,*n*+2_), where *t*_*i*,*n*+1_ = 1, *t*_*i*,*n*+2_ = −(1/2)∑_*w*=1_^*n*^ *t*_*iw*_^2^ and *t*_*j*,*n*+1_ = 1, *t*_*j*,*n*+2_ = −(1/2)∑_*w*=1_^*n*^ *t*_*jw*_^2^. After receiving *M*_*P*_, for *i*, *j* = 1,…, *m*, *i* ≠ *j*, the MD-ATM computes

(8) Δij′=MP·(Ti−Tj)T=[b11·qP1⋯b1,n+2·qP,n+2⋮⋮bk1·qP1⋯bk,n+2·qP,n+2]·[ti1−tj1⋮ti,n+2−tj,n+2].

After receiving the returned message Δ_*ij*_′, for *i*, *j* = 1,…, *m*, *i* ≠ *j*, *P* uses PMDD to compute

(9) Δij=sP·Δij′=sP·MP·(Ti−Tj)T=(sP1,…,sPk)·[b11·qP1⋯b1,n+2·qP,n+2⋮⋮bk1·qP1⋯bk,n+2·qP,n+2] ·[ti1−tj1⋮ti,n+2−tj,n+2]=∑u=1ksPu·bu1·qP1·(ti1−tj1)+⋯ +∑u=1ksPu·bu,n+2·qP,n+2·(ti,n+2−tj,n+2)=∑w=1n+2qPw·(tiw−tjw).

Thus, we have

(10) Δij=∑w=1n+2qPw·tiw−∑w=1n+2qPw·tjw=(∑w=1nqPw·tiw+qP,n+1·ti,n+1+qP,n+2·ti,n+2) −(∑w=1nqPw·tjw+qP,n+1·tj,n+1+qP,n+2·tj,n+2)=(∑w=1nqPw·tiw+qP,n+1+ti,n+2) −(∑w=1nqPw·tjw+qP,n+1+tj,n+2)=(∑w=1nqPw·tiw−12∑w=1nqPw2−12∑w=1ntiw2) −(∑w=1nqPw·tjw−12∑w=1nqPw2−12∑w=1ntjw2)=12[∑w=1n(qPw−tjw)2−∑w=1n(qPw−tiw)2]=12(distqP↔tj2−distqP↔ti2).

Obviously, if for all *j* = 1,…, *m*, *j* ≠ *z*, Δ_*zj*_ = (1/2)(dist⁡_*q*_*P*_↔*t*_*j*__^2^ − dist⁡_*q*_*P*_↔*t*_*z*__^2^) > 0, then *t*_*z*_, *z* ∈ {1,…, *m*}, is the trait vector of the diagnosed disease. The report *r*_*z*_ corresponding to the trait vector *t*_*z*_ is the diagnosed report. Taking the index *z* as the input of the following OT_1_^*m*^ protocol, the patient can finally get the disease report *r*_*z*_ from the set (*r*_1_, *r*_2_,…, *r*_*m*_) of the database.

Therefore, our scheme is correct.

### 5.2. Security

In this subsection, we strictly prove the security of our scheme. From the whole process, we can specify that the two parties in our system are the patient *P* and the hospital. They cooperate to compute the function *f*(*q*_*P*_, (*d*_1_, *d*_2_,…, *d*_*m*_)) = *r*_*z*_, where *r*_*z*_ is the disease diagnostic report corresponding to the disease *d*_*z*_ and the distance dist⁡_*q*_*p*_↔*t*_*z*__ satisfies the condition dist⁡_*q*_*p*_↔*t*_*z*__^2^ = min⁡{dist⁡_*q*_*p*_↔*t*_*j*__^2^}_*j*=1,…,*m*_. As mentioned in Section 3, we should achieve two security goals, that is, keeping both parties' inputs private. We apply the real-ideal simulation paradigm to prove that our scheme has achieved the two goals in the scenarios of semihonest adversaries assuming the OT_1_^*m*^ protocol we used is secure.

**Theory 1.** *Our privacy-preserving self-helped medical diagnosis scheme is secure against semihonest adversaries if the *OT_1_^*m*^ *protocol is secure.*

**Proof**

Notice that the view of *P*_*i*_, {VIEW_*i*_(*q*_*p*_, {*d*_1_,…, *d*_*m*_})}_*i*=1,2_, in the real execution consists of three parts, the private input, random tape, and the messages received from the other party including the output. Therefore, we can get the views of *P*_1_ and *P*_2_, respectively, in the real execution as follows:

(11) {VIEW1(qp,{di}i=1,…,m)} ={qP,sP,BP,{Δij′}i,j=1,…,m,i≠j,   {VIEW1OT1m(z,{ri}i=1,…,m)},rz};

(12) {VIEW2(qp,{di}i=1,…,m)} ={{di}i=1,…,m,MP,{VIEW2OT1m(z,{ri}i=1,…,m)},λ},

where {VIEW_1_^OT_1_^*m*^^(*z*, {*r*_*i*_}_*i*=1,…,*m*_)} and {VIEW_2_^OT_1_^*m*^^(*z*, {*r*_*i*_}_*i*=1,…,*m*_)} are the views produced in the execution of OT_1_^*m*^ protocol.

From the definition of security, we need to construct the probabilistic polynomial-time algorithm *S*_1_/*S*_2_ so that given the input and output of the patient *P*_1_/the hospital *P*_2_, (*q*_*P*_; *r*_*z*_)/({*d*_*i*_}_*i*=1,…,*m*_; *λ*), it can output a simulated view {*S*_1_(*q*_*P*_; *r*_*z*_)}/{*S*_2_({*d*_*i*_}_*i*=1,…,*m*_; *λ*)}, which is indistinguishable to the view {VIEW_1_(*q*_*p*_, {*d*_*i*_}_*i*=1,…,*m*_)}/{VIEW_2_(*q*_*p*_, {*d*_*i*_}_*i*=1,…,*m*_)} in the real execution of the scheme; that is,

(13) $$\begin{array}{c} \left\{S_{1}\left(q_{P};r_{z}\right)\right\}≅\left\{{\text{VIEW}}_{1}\left(q_{p},{\left\{d_{i}\right\}}_{i=1,\ldots ,m}\right)\right\},\\ \left\{S_{2}\left({\left\{d_{i}\right\}}_{i=1,\ldots ,m};\lambda \right)\right\}≅\left\{{\text{VIEW}}_{2}\left(q_{p},{\left\{d_{i}\right\}}_{i=1,\ldots ,m}\right)\right\}. \end{array}$$

In the following discussion, we follow the real-ideal simulation paradigm to construct such probabilistic polynomial-time algorithms *S*_1_, *S*_2_. We separately prove the case when *P*_2_ is semihonest and when *P*_1_ is semihonest.

*Case 1* (*P*_2_ is semihonest). In this case, we only need to construct a simulator *S*_2_ so that, given *P*_2_'s input {*d*_*i*_}_*i*=1,…,*m*_ and output *λ*, *S*_2_ can simulate *P*_2_'s view in the real execution presented above as (12).

Firstly, since we assume that the OT_1_^*m*^ protocol used in our scheme is secure and can be taken as a black-box, there exists an algorithm *S*_2_^OT_1_^*m*^^: given the input {*r*_*i*_}_*i*=1,…,*m*_ and the output *λ*, it can simulate *P*_2_'s view of the OT_1_^*m*^ execution and output {*S*_2_^OT_1_^*m*^^({*r*_*i*_}_*i*=1,…,*m*_; *λ*)} so that

(14) $$\begin{array}{c} \left\{S_{2}^{\text{O}{\text{T}}_{1}^{m}}\left({\left\{r_{i}\right\}}_{i=1,\ldots ,m};\lambda \right)\right\}≅\left\{{\text{VIEW}}_{2}^{\text{O}{\text{T}}_{1}^{m}}\left(z,{\left\{r_{i}\right\}}_{i=1,\ldots ,m}\right)\right\}. \end{array}$$

Next, notice that *S*_2_ is given ({*d*_*i*_}_*i*=1,…,*m*_; *λ*); it can easily simulate the remaining parts of (12) by randomly choosing a *k* × (*n* + 2) matrix *M* which is indistinguishable to the blinded matrix *M*_*P*_. Then, *S*_2_ outputs the simulated view,

(15) {S2({di}i=1,…,m;λ)} ={{di}i=1,…,m,M,{S2OT1m({ri}i=1,…,m;λ)},λ}.

Obviously, we can conclude that

(16) $$\begin{array}{c} \left\{S_{2}\left({\left\{d_{i}\right\}}_{i=1,\ldots ,m};\lambda \right)\right\}≅\left\{{\text{VIEW}}_{2}\left(q_{p},{\left\{d_{i}\right\}}_{i=1,\ldots ,m}\right)\right\}. \end{array}$$

*Case 2* (*P*_1_ is semihonest). Similar to Case 1, we only need to construct a simulator *S*_1_ so that given *P*_1_'s input *q*_*P*_ and output *r*_*z*_, *S*_1_ can simulate *P*_1_'s view in the real execution presented above as (11).

As discussed above, since the OT_1_^*m*^ protocol is secure, there exists an algorithm *S*_1_^OT_1_^*m*^^: given the input *z* and the output *r*_*z*_, it can simulate *P*_1_'s view of the OT_1_^*m*^ execution and output {*S*_1_^OT_1_^*m*^^(*z*; *r*_*z*_)} so that

(17) $$\begin{array}{c} \left\{S_{1}^{\text{O}{\text{T}}_{1}^{m}}\left(z;r_{z}\right)\right\}≅\left\{{\text{VIEW}}_{1}^{\text{O}{\text{T}}_{1}^{m}}\left(z,{\left\{r_{i}\right\}}_{i=1,\ldots ,m}\right)\right\}. \end{array}$$

Next, given (*q*_*P*_; *r*_*z*_), *S*_1_ then simulates the remaining parts of *P*_1_'s view in the real execution as follows.

Firstly, as in the real execution, *S*_1_ extends the original health data from an *n*-vector *q*_*P*_ = (*q*_*P*1_,…, *q*_*Pn*_) to an (*n* + 2)-vector *Q*_*P*_ = (*q*_*P*1_,…, *q*_*Pn*_, *q*_*P*,*n*+1_, *q*_*P*,*n*+2_), where *q*_*P*,*n*+1_ = −(1/2)∑_*w*=1_^*n*^ *q*_*Pw*_^2^ and *q*_*P*,*n*+2_ = 1. Then, *S*_1_ randomly chooses a password *s*_*P*_′ = (*s*_*P*1_′,…, *s*_*Pk*_′) and then generates a *k* × (*n* + 2) matrix

(18) $$\begin{array}{c} B_{P}^{\prime }=\left[\begin{array}{ccc} b_{11}^{\prime } & \cdots & b_{1,n+2}^{\prime }\\ \vdots & & \vdots \\ b_{k1}^{\prime } & \cdots & b_{k,n+2}^{\prime } \end{array}\right], \end{array}$$

where ∑_*u*=1_^*k*^ *s*_*Pu*_′ · *b*_*uw*_′ = 1, *w* = 1,…, *n* + 2. By blinding *Q*_*P*_ using the matrix *B*_*P*_′, *S*_1_ further extends *Q*_*P*_ to a matrix

(19) $$\begin{array}{c} M_{P}^{\prime }=\left[\begin{array}{ccc} b_{11}^{\prime }\cdot q_{P1} & \cdots & b_{1,n+2}^{\prime }\cdot q_{P,n+2}\\ \vdots & & \vdots \\ b_{k1}^{\prime }\cdot q_{P1} & \cdots & b_{k,n+2}^{\prime }\cdot q_{P,n+2} \end{array}\right]. \end{array}$$

Then *S*_1_ randomly selects *m* vectors, *T*_*j*_′ = (*t*_*j*1_′,…, *t*_*jn*_′, *t*_*j*,*n*+1_′, *t*_*j*,*n*+2_′), *t*_*j*,*n*+1_′ = 1, *t*_*j*,*n*+2_′ = −(1/2)∑_*w*=1_^*n*^ *t*_*jw*_^′2^, *j* = 1,…, *m*, and for all *j* = 1,…, *m*, *j* ≠ *z*, ∑_*w*=1_^*n*^(*q*_*Pw*_ − *t*_*zw*_′)^2^ < ∑_*w*=1_^*n*^(*q*_*Pw*_ − *t*_*jw*_′)^2^; otherwise, *S*_1_ reselects *T*_*j*_′, *j* = 1,…, *m*. Afterwards, *S*_1_ computes Λ_*ij*_′ = *M*_*P*_′ · (*T*_*i*_′ − *T*_*j*_′)^*T*^, *i*, *j* = 1,…, *m*, *i* ≠ *j*. Thus, for *i*, *j* = 1,…, *m*, *i* ≠ *j*,   Λ_*ij*_ = *s*_*P*_′ · Λ_*ij*_′ = ∑_*w*=1_^*n*+2^ *q*_*Pw*_ · (*t*_*iw*_′ − *t*_*jw*_′).

Therefore, we have

(20) Λij=∑w=1n+2qPw·tiw′−∑w=1n+2qPw·tjw′=(∑w=1nqPw·tiw′+qP,n+1·ti,n+1′+qP,n+2·ti,n+2′) −(∑w=1nqPw·tjw′+qP,n+1·tj,n+1′+qP,n+2·tj,n+2′)=(∑w=1nqPw·tiw′+qP,n+1+ti,n+2′) −(∑w=1nqPw·tjw′+qP,n+1+tj,n+2′)=(∑w=1nqPw·tiw′−12∑w=1nqPw2−12∑w=1ntiw′2) −(∑w=1nqPw·tjw′−12∑w=1nqPw2−12∑w=1ntjw′2)=12[∑w=1n(qPw−tjw′)2−∑w=1n(qPw−tiw′)2]=12(distqP↔tj′2−distqP↔ti′2).

Since for all *j* = 1,…, *m*, *j* ≠ *z*, we have ∑_*w*=1_^*n*^(*q*_*Pw*_ − *t*_*zw*_′)^2^ < ∑_*w*=1_^*n*^(*q*_*Pw*_ − *t*_*jw*_′)^2^. Obviously, for all *j* = 1,…, *m*, *j* ≠ *z*, Λ_*zj*_ = (1/2)(dist⁡_*q*_*P*_↔*t*_*j*_′_^2^ − dist⁡_*q*_*P*_↔*t*_*z*_′_^2^) > 0 and *t*_*z*_, *z* ∈ {1,…, *m*}, is the trait vector of the diagnosed disease. The report *r*_*z*_ corresponding to the trait vector *t*_*z*_ is the diagnosed report, which matches the relationship in the real execution.

Now, *S*_1_ can output the simulated view,

(21) {S1(qP;rz)} ={qP,sP′,BP′,{Λij′}i,j=1,…,m,i≠j,{S1OT1m(z;rz)},rz}.

Since *s*_*P*_′ and *B*_*P*_′ are randomly chosen and ∑_*u*=1_^*k*^ *s*_*Pu*_′ · *b*_*uw*_′ = 1, *w* = 1,…, *n* + 2, as *s*_*P*_ and *B*_*P*_ in the real view {VIEW_1_(*q*_*p*_, {*d*_*i*_}_*i*=1,…,*m*_)}. Due to the randomness and relationship, it is easy to find that (*s*_*P*_′, *B*_*P*_′) is indistinguishable to (*s*_*P*_, *B*_*P*_). From the construction process of {Λ_*ij*_′}_*i*,*j*=1,…,*m*,*i*≠*j*_, it is obvious to conclude that {Λ_*ij*_′}_*i*,*j*=1,…,*m*,*i*≠*j*_ is indistinguishable to the set {Δ_*ij*_′}_*i*,*j*=1,…,*m*,*i*≠*j*_. Combined with (17), we have

(22) $$\begin{array}{c} \left\{S_{1}\left(q_{P};r_{z}\right)\right\}≅\left\{{\text{VIEW}}_{1}\left(q_{p},{\left\{d_{i}\right\}}_{i=1,\ldots ,m}\right)\right\}. \end{array}$$

## 6. Conclusions

In this paper, we consider the problem of how to securely make diagnosis without leaking patient's health data, diagnosed result, and hospital's disease database in wireless sensor networks. By applying the idea of secure two-party computation and the technology of oblivious transfer, we propose a privacy-preserving self-helped medical diagnosis scheme so that patients can privately diagnose themselves by inserting a health card into a self-helped MD-ATM to obtain the diagnostic report just like drawing money from a bank ATM. We also have a detailed analysis about the correctness and further strictly prove the security following the real-idea simulation paradigm. We expect to provide people another perspective on future medical care.

## Figures and Tables

**Algorithm 1:**
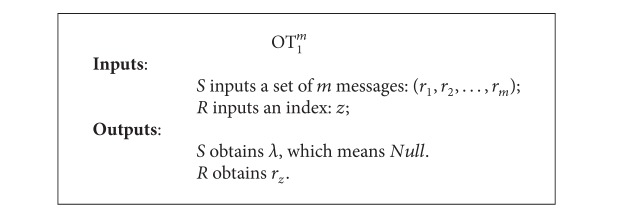


## References

[1] Wan J, Ullah S, Lai C (2013). Cloud-enab led wireless body area networks for pervasive healthcare. *IEEE Network*.

[2] Doukas C, Pliakas T, Maglogiannis I Mobile healthcare information management utilizing Cloud Computing and Android OS.

[3] Yuan J, Yu S Efficient privacy-preserving biometric identification in cloud computing.

[4] Liu J, Wang Q, Wan J, Xiong J, Zeng B (2013). Towards key issues of disaster aid based on wireless body area networks. *KSII Transactions on Internet and Information Systems*.

[5] Al Ameen M, Liu J, Kwak K (2012). Security and privacy issues in wireless sensor networks for healthcare applications. *Journal of Medical Systems*.

[6] Schwartzand PM, Reidenberg JR (1996). *Data Privacy Law: A Study of United States Data Protection*.

[7] Nissenbaum H (2010). *Privacy in Context: Technology, Policy, and the Integrity of Social Life*.

[8] Gentry C *A fully homomorphic encryption scheme [doctoral dissertation]*.

[9] Brakerski Z, Vaikuntanathan V Efficient fully homomorphic encryption from (standard) LWE.

[10] López-Alt A, Tromer E, Vaikuntanathan V On-the-fly multiparty computation on the cloud via multikey fully homomorphic encryption.

[11] Bringer J, Chabanne H, Patey A (2013). Privacy-preserving biometric identification using secure multiparty computation: an overview and recent trends. *IEEE Signal Processing Magazine*.

[12] Yao AC Protocols for secure computations.

[13] Goldreich OS, Mical S, Wigderson A How to play any mental game.

[14] Goldreich OS Secure multiparty computation.

[15] Goldreich O (2004). *Foundations of Cryptography: Volume 2, Basic Applications*.

[16] Prabhakaran MM, Sahai A (2013). *Secure Multiparty Computation*.

[17] Chaum D, Crepeau C, Damgard I Multi-party unconditionally secure protocols (extended abstract).

[18] Damgard I, Pastro V, Smart NP, Zakarias S (2012). Multiparty computation from somewhat homomorphic encryption. *Advances in Cryptology—Crypto 2012*.

[19] Lindell Y, Pinkas B (2007). An efficient protocol for secure two-party computation in the presence of malicious adversaries. *Advances in Cryptology—EUROCRYPT 2007*.

[20] Pinkas B, Schneider T, Smart NP, Williams SC (2009). Secure two-party computation is practical. *Advances in Cryptology—ASIACRYPT 2009*.

[21] Rabin MO (1981). How to exchange secrets b y oblivious transfer.

[22] Even S, Goldreich O, Lempel A (1985). A randomized protocol for signing contracts. *Communications of the ACM*.

[23] Naor M, Pinkas B (1999). Oblivious transfer with adaptive queries. *Advances in Cryptology—CRYPTO’ 99*.

